# Minimally invasive, transapical dual-lumened cannula for short-term left ventricular support in a clinically relevant large animal model^†^

**DOI:** 10.1093/ejcts/ezaf173

**Published:** 2025-05-21

**Authors:** Marcell Székely, László Székely, Mónika C. Dénes, Tamás Radovits, Béla Merkely, István Hartyánszky

**Affiliations:** Department of Cardiothoracic and Vascular Surgery, Central Hospital of Northern Pest - Military Hospital, Budapest, Hungary; Heart and Vascular Center, Semmelweis University, Budapest, Hungary; Department of Cardiothoracic and Vascular Surgery, Central Hospital of Northern Pest - Military Hospital, Budapest, Hungary; Department of Cardiothoracic and Vascular Surgery, Central Hospital of Northern Pest - Military Hospital, Budapest, Hungary; Heart and Vascular Center, Semmelweis University, Budapest, Hungary; Heart and Vascular Center, Semmelweis University, Budapest, Hungary; Heart and Vascular Center, Semmelweis University, Budapest, Hungary

**Keywords:** Mechanical circulatory support, Cardiogenic shock, Antegrade flow

## Abstract

**OBJECTIVES:**

Cardiogenic shock is still a major clinical challenge, despite the available devices. We developed a minimally invasive, transapical dual-lumened cannula, which can provide antegrade circulatory support and unloading for the left ventricle (LV). After using 3D printing technology, we wanted to test whether our prototypes are haemodynamically competent and implantable in an experimental large porcine model as a proof of concept study.

**METHODS:**

We implanted our cannula prototypes to 7 healthy porcines via median sternotomy (*n* = 6) and via minimally invasive access (*n* = 1), transapically, under fluoroscopic control, off-pump. The cannulas were connected to a heart-lung machine, and we went from 2.5, to 3.5, 4, 4.5, 5 and 5.5 l/min flow with 15–15 min intervals on each flow to ensure LV support. Different metabolic and haemodynamic parameters were continuously monitored.

**RESULTS:**

Implantation time was 14 ± 5 min. The cardiac output of the right ventricle elevated with the LV and roller pump performance from baseline of 4.81 ± 2.09 to 6.17 ± 1.02 l/min at 5.5 l/min pump flow. Mean arterial pressure and central venous pressure changed from 68.9 ± 9.4 and 9.2 ± 2.4 mmHg, to 72.8 ± 11.3 and 9.8 ± 3 mmHg, respectively. Serum lactic acid and other metabolic parameters were not changed significantly.

**CONCLUSIONS:**

We have successfully proved in a large animal study that our prototypes are implantable and can provide up to 5.5 l/min cardiac output. They could assist, then fully replace the function of the LV using a roller pump during our study. Further investigations are planned in the future using centrifugal pumps for longer-term support.

## GRAPHICAL ABSTRACT

**Figure:**
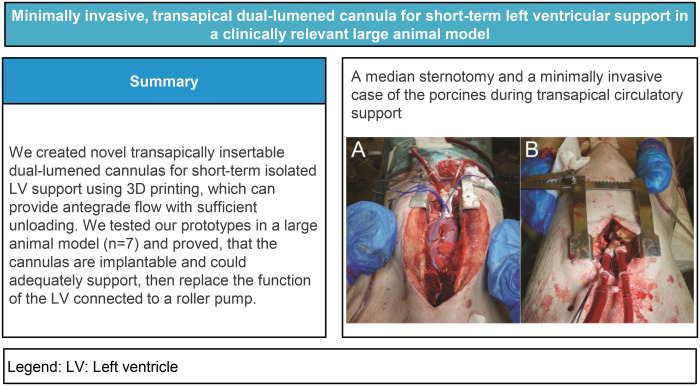

## INTRODUCTION

Cardiogenic shock (CS) is a well-known clinical entity, most commonly caused by acute myocardial infarction, myocarditis, cardiotomy or acute onset of chronic heart failure. The treatment of CS remains to be a major clinical challenge with an in-hospital mortality about 40% [1, 2]. After the failure of pharmacological therapy, mechanical circulatory support (MCS) devices are the only possibilities to overcome CS [2, 3]. These percutaneous or surgical devices have several important complications due to their use and working method affecting morbidity and mortality [3, 4]. During the past decade, minimally invasive techniques for surgical devices are getting more attention, either for long-term or short-term support. Sufficient flow (antegrade) and durability are considered to be advantages, particularly when median sternotomy can be avoided [4].

In this proof-of-concept study, we present our experiences with our transapically insertable dual-lumened cannula prototype in large experimental pigs. We published the first steps for creating and planning this cannula earlier, thereby we would only like to publish our further studies evaluating this concept. The important factors, which were considered in the sizing and planning, are also highlighted there [5]. After analysing CT angiography scans of end-stage heart failure patient’s left ventricle (LV) and aortic root dimensions which we defined in our previous study, we modified our cannula prototypes to be feasible for *in vivo* porcine studies, keeping the important dimensions similar to real human cases [5]. It was a main goal for us to design the cannulas in a way that they can possibly be implanted minimal invasively through the apex of the heart, besides providing sufficient unloading and up to 5.5 l/min antegrade flow to support the LV using an extracorporeal pump. The cannulas were created by using 3D printing technology, to have the possibility to make them with patient-specific sizes.

## MATERIALS AND METHODS

### Cannula design and manufacturing

**Figure 1:**
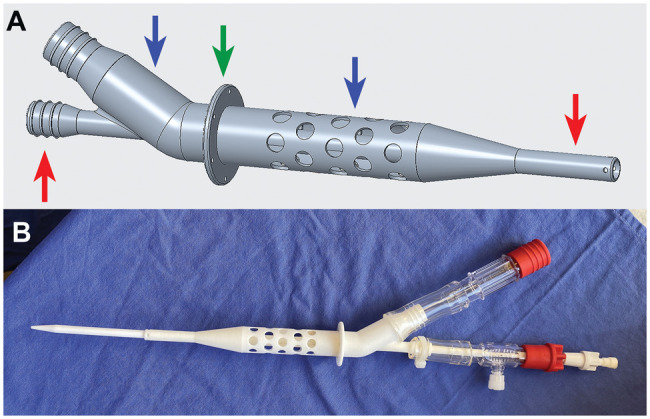
The model of the dual-lumened cannula prototype in a 3D planning software (**A**) and a printed model with additional tubing (**B**). In part A, the red arrows are pointing at the inner tube with its apertures at the tip. The blue arrows are pointing at the outer drainage tube with its draining apertures and conical ending where the outer wall of the drainage tube goes into the outer wall of the inner tube. The green arrow is pointing at the skirt which supposed to be at the outer wall of the LV. The holes in this skirt are placed to guide the apical sutures for stabilizing the position of the cannula.

All our dual-lumened cannula prototypes were printed using PA2200 biocompatible material and an EOS P396 printer (EOS GMBH, Krailling, Germany) (Fig. 1). PTC Creo software (PTC, Boston, USA) was used for designing. Our dual-lumened cannula is a tube-in-tube solution (co-axial fashion) using the outer drainage tube (50.4 French) and the thinner inner outflow tube (20 French) which are separated completely from each other. The outer drainage tube ensures oxygenated blood flow from the LV to an extracorporeal pump, and then the inner longer outflow tube guides the blood through the aortic valve above the coronary artery ostias. This system provides systemic and coronary blood supply, therefore supports LV function. Within the lumen of the drainage tube, we designed spacers to ensure luminal stability. The outer wall runs conically into the wall of the inner tube. When the cannula is properly positioned in the left heart, the drainage apertures are designed to end before the mitral valve anterior leaflet could be possibly sucked into the highest aperture. The skirt of the cannula should be at the epicardial wall of the apex. From the skirt, the length of the inner and outer tube without the conical ending is 139 mm and 51 mm. The first line of apertures starts 5.8 mm from the skirt with 5.4 mm diameters. The side openings at the tip of the inner tube are for coronary perfusion. Other tools in the dual-lumened cannula, such as dilators, tubing, tubing connectors (made from PVC) and taps for contrast agent, were necessary to perform implantations using Seldinger-technique.

### *In vivo* porcine evaluation

The experiments were carried out in accordance with EU Directive 2010/63 for the protection of animals used for scientific purposes and in compliance with criteria set down in the US National Institutes of Health Guidelines for the Care and Use of Laboratory Animals. The study was reviewed and approved by the Animal Use and Care Committee of Semmelweis University Budapest and by the Ethical Committee of Hungary for Animal Experimentation (permission number from the Government Office for Pest County, Hungary: PE/EA/00241-2/2023).

All experiments were done at the animal lab of Semmelweis University. The insertion and performance of our dual-lumened cannula prototypes with a heart-lung machine were tested in 7 mature porcines with a 100% success rate. The porcines are considered to be healthy regarding their cardiovascular condition. They were last fed 1 day before the operations.

### Measurements, instrumentation and anaesthesia

The animals were sedated by an im. injection of 25 mg/kgBW ketamine (50 mg/ml, Gedeon Richter Plc. Budapest, Hungary) and 0.3 mg/kgBW midazolam (15 mg/3 ml, Kalceks AS, Riga, Latvia) and were carefully transported into the laboratory. Anesthesia was induced by 2.5 mg/kgBW iv. propofol (10 mg/ml, Fresenius Kabi, Bad Homburg, Hessen, Germany) injection into the ear vein, followed by orotracheal intubation with an endotracheal tube, and then controlled ventilation was applied (Dräger Primus, Drägerwerk AG & Co, Lübeck, Schleswig-Holstein, Germany). Analgesia was provided by an im. injection of 0.3 mg/kgBW butorphanol (10 mg/ml Nalgosed, Bioveta, Czech Rep.) and local infiltration with lidocaine (1.5 ml). Maintenance of anaesthesia was reached by the use of isoflurane (1.5–3%) and propofol, dosed by keeping normal arterial blood pressure (ABP) and heart rate (HR). A 12-lead ECG was recorded of the porcines after they entered to the operating room and throughout each operation until euthanasia. We surgically exposed the left femoral artery and vein, where we introduced a high-fidelity microtip pressure catheter (Millar Instruments, Houston, TX, USA) to the artery for continuous ABP monitoring, measured as systolic and mean arterial pressure (SAP, MAP). The right external jugular vein and common carotid artery was exposed and cannulated to gain venous and arterial blood samples and introduce a central venous catheter to continuously monitor central venous pressure (CVP) and create a route for drug therapy and fluid administration. After sternotomy and pericardiotomy, we placed an ultrasound flow probe on the pulmonary trunk connected to a TS420 perivascular flow meter (Transonic, Ithaca, NY, USA) to monitor cardiac output (CO). Before cannulation and initiation of MCS ,therapy, we obtained the first arterial and venous blood samples, such as pH, PaCO_2_, PaO_2_, PvCO_2_, PvO_2_, potassium, sodium, haematocrit, haemoglobin and base excess (BE) (blood gas test) besides blood glucose. Pulse oximetry was continuously monitored via the tongue or ear of the animals. We defined the measured parameters before cannulation as baseline characteristics. After transapical cannulation, we obtained arterial (oxygenated) blood samples after 15 min of each provided flow (2.5, 3.5, 4, 4.5, 5, 5.5 l/min) by the heart-lung machine, practically at the end of the time intervals. We obtained the venous blood samples at baseline and at maximum pump flow. The tubes of the heart-lung machine were filled with 1.5 l crystalloid Ringer-lactate prime volume and 100 ml 20% mannitol; therefore, haemodilution of the porcines thorough the operations was expected as long as subsequent changes in their blood gas samples.

### Surgical exposure, cannula insertion and MCS management

**Figure 2:**
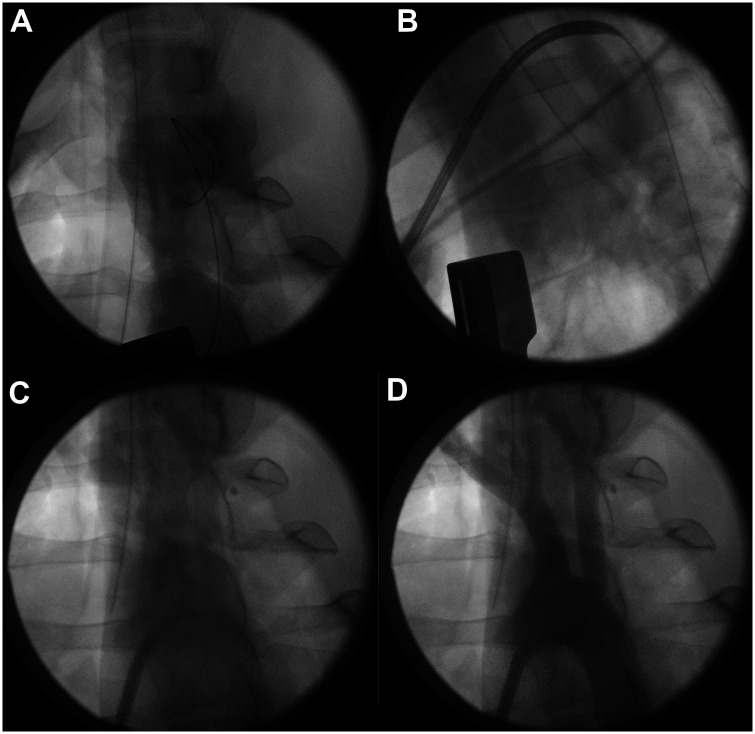
Fluoroscopic images of the different steps of cannula insertion and ideal positioning. In part **A**, a guidewire can be seen, which was inserted through the LV apex to the aortic root, and descending aorta. In part **B**, a dilator in the inner tube can be seen in the ascending aorta and aortic arch. Injected contrast agent to the inner tube, after dilator removement, where the aortic arch and its branches can be seen as in parts **C** and **D.**

**Figure 3:**
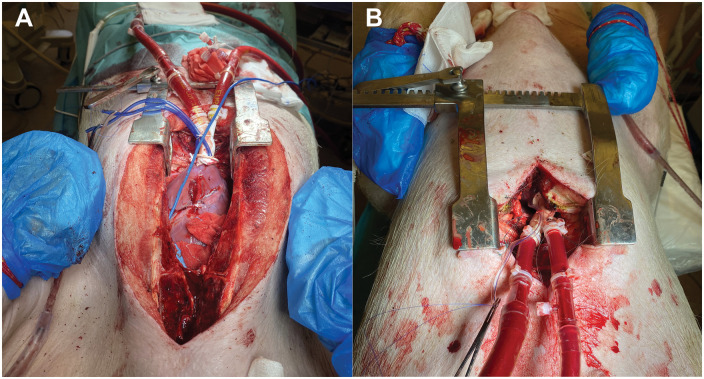
Transapical circulatory support systems using median sternotomy (part **A**) and minimally invasive subxiphoidal access (part **B**). In part A, you can see the apical pledgeted purse-string sutures which stabilized the cannulas with turniquets. A blue cable goes to the perivascular flow probe placed on the pulmonary trunk.

We performed complete median sternotomy on the 7 porcines including that 1 case where we started with a 6 cm minimally invasive incision subxiphoidally (Videos 1 and 2). In that 1 case, we inserted the dual-lumened cannula transapically through this small incision, confirmed its ideal place with angiography and then we completed median sternotomy to place the flow probe on the pulmonary trunk for complete measurements (Video 2). The minimally invasive case was done to evaluate the feasibility of insertion through a significantly smaller incision of our cannula concept. After median sternotomy was accomplished (regarding the other cases), we opened the pericardium and placed the flow probe on the pulmonary trunk and defined baseline characteristics of the animals. Two apical pledgeted purse-string sutures were made. We positioned a C-arm at the porcine’s thorax, and after iv. heparinization, we introduced a guidewire through the apex, crossed the aortic valve and went down to the descending aorta under fluoroscopic control (Phillips BV Pulsera C-arm) (Fig. 2). We cannulated transapically using Seldinger-technique with our prototype to avoid rupture of the apical myocardium. Using the guidewire, we correctly positioned the cannulas. As the next step, the guidewire and the dilator were removed, and we confirmed the position of the cannula with contrast agent under fluoroscopy which we injected through the inner tube (Fig. 2). The cannula’s inner and outer tubes were connected to the tubing of the heart-lung-machine (Stöckert S3, LivaNova PLC, London, UK), and we started LV support with 2.5 l/min (Fig. 3, Video 1, Video 2). After 15–15 min on each flow provided by the pump, we elevated the support to 3.5, 4, 4.5, 5 and finally to 5.5 l/min. Before elevating the flow, blood samples were taken. In the blood samples, lactic acid was measured in all cases as a parameter referring to perfusion adequacy. We used the oxygenator, reservoir, bubble traps, roller pump and the yellow sucker of the circuit (LivaNova PLC, London, UK). When we reached 15 min of perfusion with a 5.5 l/min pump flow, pumping was stopped, decannulation was performed with adequate closure of the apical wound, and the porcines were euthanized (Video 1).

### Animal management during the operation

After the introduction of central venous catheter, Ringer-lactate was administered when needed to titrate CVP level between 3 and 10 mmHg. Before transapical cannulation, iv. heparin was administered (300 U/kg) until activated clotting time was above 480 s, which was measured with an ACT Plus device (Medtronic, MN, USA) and kaolin was used to activate clotting. If glucose level was below the acceptable level (<4 mmol/l), we supplemented glucose iv. During the operations, we used noradrenaline to maintain MAP > 60 mmHg if required.

### Data collection and analysis

Signals from the previously mentioned haemodynamic measurements (MAP, SBP, CVP, CO, ECG) were continuously recorded, stored and displayed with the LabChart 8 software on a personal computer connected to a PowerLab system (ADInstruments, Colorado Springs, CO, USA). Blood was sampled for complete blood counts, before MCS support and at terminating besides previously described tests. Continuous data were reported as mean with standard deviation. Statistical analysis was performed using SPSS 28.0.1.0 (IBM, Armonk, NY, USA) software. To compare mean baseline and maximum flow values, we used 2-tailed paired t-test. We considered a *P*-value of <0.05 statistically significant.

### Study termination

After experiments were finished, all animals were euthanized with iv. administration of overdosed pentobarbital and saturated KCl solutions (75–100 ml), confirmed by ECG monitoring. We performed necropsy on each heart, where we opened the LV and aortic root from the apical incision. The LV walls, chordae, mitral valve apparatus, aortic root and aortic valves were macroscopically visualized whether there is any injury on the different structures.

## RESULTS

The dual-lumen cannula prototypes were successfully created, printed and investigated in 7 porcines as a part of an LV MCS system. We successfully inserted our cannulas transapically using Seldinger-technique regarding all operations either from median sternotomy or a minimally invasive approach without causing any intracardiac injury, arrhythmia or significant bleeding (>500 ml). We did not experience any device thrombus formation in the explanted cannulas. The prototypes were ideally positioned during all procedures; the tip of the inner tube was in the ascending aorta beyond the aortic valve, and the apertures of the outer draining tube were in the LV in all cases. With additional blade incision during implantation, all cannulas went into the LV and then to the aorta quiet easily; therefore, bleeding from the apertures was not a significant issue regarding the implantation. Measurements were complete at all experiments. Signs of myocardial ischaemia on the ECG could not be seen.

Baseline characteristics of the porcines can be seen in Tables 1 and 2, and Supplementary Material 1. The mean mass of the animals was 96.6 ± 4.3 kg, and the body surface area was 1.75 ± 0.05 m^2^. On the ECG, we could confirm that all porcines had sinus rhythm before cannulation. After cannulation and the start of circulatory support, the haemodynamic and haematological changes we experienced at different pump flows can be seen in Tables 1 and 2. As we expected (because the priming volume), haemodilution occurred. The transapical cannulation time from the start of the apical purse-string sutures was 14 ± 5 min. From median sternotomy until the euthanasia, we let the animals cooled by themselves from 38.6 ± 0.97 °C to 37 ± 0.42 °C.

**Table 1:** Haematology

	Baseline (no pump flow)	At 5.5 l/min pump flow	Δ 5.5 l/min-baseline	*P*-value
Hb (g/dl)	11.5 ± 3.2	7.8 ± 1.9	−3.7 ± 2	0.058
WBC (10^3^/micro l)	17.8 ± 2.8	11.8 ± 5.2	−6 ± 6.6	0.061
RBC (million/micro l)	6.5 ± 1.8	4.3 ± 1.1	−2.1 ± 0.9	0.053
Platelet count (G/l)	295.2 ± 112.4	191.2 ± 103.2	−104 ± 127.4	0.166
Lymphocytes (%)	55.6 ± 6.3	38.9 ± 12.4	−16.7 ± 12.3	**0.037**
Granulocytes (%)	46.4 ± 13.8	59.6 ± 12.8	13.2 ± 14.7	0.155
Haematocrit (%)	29.9 ± 4.4	22.7 ± 4.7	−7.1 ± 6.5	**0.012**
Expected haematocrit (haemodilution)	29.9 ± 4.4	19.7 ± 2.9 (haematocrit*2/3)	−10.1 ± 1.5	**<0.01**

*P* < 0.05 values are highlighted in bold; values are expressed as mean ± standard deviation.

Hb: haemoglobin; RBC: red blood cells; WBC: white blood cells; Δ: Difference between values at 5.5 l/min and baseline values.

**Table 2:** Haemodynamics

	CO (l/min)	Lactic acid (mmol/l)	CVP (mmHg)	MAP (mmHg)	HR (1/min)
Baseline (no pump flow)	4.81 ± 2.09	2.30 ± 1.61	9.2 ± 2.4	68.9 ± 9.4	79 ± 17
2.5 l/min	4.95 ± 1.10	1.94 ± 0.81	9.4 ± 2.7	58.8 ± 5.2	90 ± 22
3.5 l/min	5.29 ± 1.21	2.50 ± 1.10	9.5 ± 2.8	61.5 ± 9.8	90 ± 15
4 l/min	5.40 ± 1.32	2.83 ± 1.53	9.7 ± 3.1	61.9 ± 5.2	90 ± 21
4.5 l/min	6.07 ± 1.12	2.91 ± 1.75	10.4 ± 3.3	64.7 ± 8.3	86 ± 15
5 l/min	5.92 ± 0.81	2.93 ± 1.75	10.8 ± 4.3	66.9 ± 11.3	88 ± 16
5.5 l/min	6.17 ± 1.02	3.12 ± 1.88	9.8 ± 3	72.8 ± 11.3	99 ± 20
Δ 5.5 l/min-baseline (*P*-value)	1.51 ± 1.2 (0.09)	0.6 ± 1.97 (0.52)	2 ± 3 (0.32)	2.4 ± 12.3 (0.72)	18 ± 19 (0.08)

Values are expressed as mean±standard deviation.

CO: cardiac output; CVP: central venous pressure; HR: heart rate; MAP: mean arterial pressure.

The MCS with the dual-lumened cannula has successfully assisted the LV providing flows 2.5–5.5 l/min. Oxygenation was adequate via both mechanical ventilation and the oxygenator of the pump. Other metabolic parameters changed similarly to other cardiac on-pump procedures (Supplementary Material 1). MAP, CO and CVP increased (baseline - 5.5 l/min) from 68.9 ± 9.4 to 72.8 ± 11.3 mmHg, 4.81 ± 2.09 to 6.17 ± 1.02 l/min and 9.2 ± 2.4 to 9.8 ± 3 mmHg, respectively.

## DISCUSSION

Our transapically implantable, 3D printed dual-lumened cannula prototypes were successfully implanted via median sternotomy and evaluated for short-term support using a heart-lung machine (roller pump) in 7 porcines, including that 1 case where the insertion was performed via a minimally invasive access. A heart-lung machine was used with regular components to manage potential hyper- or hypovolaemia during support and also to reach the aimed 5.5 l/min support flow; therefore, support was not inflow-dependent. We proved that our cannula and cannulation concept is feasible for short-term LV support. The deployment were relatively easy at all cases, and the cannulas were inserted using Seldinger-technique under fluoroscopic control to their ideal position reliably without causing any cardiac tissue trauma (LV, aortic root) or malignant arrythmia. The cannulas and the extracorporeal pump could successfully support, then replace LV function by producing 2.5–5.5 l/min pump flows for our 1.5 h study period (Table 2). Oxygenation of the 7 porcines was satisfactory during all operations (Supplementary Material 1). Significant change could not be seen regarding PaO2. CVP, MAP and ECG did not show significant change between each flow using adequate drug and fluid therapy. The non-significant change in lactic acid through the operations shows that systemic perfusion was sufficient for the porcines (Table 2). The relative stability in CVP values during the operations supports the fact that the right ventricle of the porcines were well-functioning, because they could follow the increased circulatory requirement produced by the LV and MCS device, as the results measured on the pulmonary trunk show us. Right ventricular distension was not observed. The elevation of the measured CO did not reach the same level as elevation in the pump-provided support comparing to baseline results. When the pump was supporting the LV with lower flows, such as 2.5 l/min, the LV drainage was not complete (partially clamped by the perfusionist); therefore, the well-functioning LV (by ejecting the remaining LV volume) and the pump together created the CO, which was relatively similar to baseline results, along with MAP, so HR did not compensatory increase on lower flows. However, when we elevated the pump flow to higher levels, such as 5.5 l/min, the LV drainage was near-complete or complete (clamp was released); therefore, CO was correlated more to the flow created by the pump and could not went higher than 6.17 ± 1.02 l/min (Table 2). In Table 1, a significant reduction in haematocrit and other blood particles can be seen, which was expected, due to the priming volume of the tubing. With this expected haemodilution, we did not experience greater reduction (2/3 of baseline) in blood particles as in regular on-pump cardiac surgical cases (Table 1).

Transapical cannulation itself is a well-known technique either for long- and short-term mechanical support, or for other procedures, even from a minimally invasive access [6, 7]. Several studies and reports have described the feasibility and safety of minimally invasive approaches, resulting in better outcomes regarding recovery, transfusion requirements and postoperative wound infections comparing to left ventricular assist device (LVAD) implantations from median sternotomy [6]. Sparing the sternum of these patients is also beneficial, especially for those who has chronic heart failure, because a later LVAD implantation or cardiac transplant can potentially occur. There are also other research groups working with a dual-lumened circulatory support concept. One from them is developing an accessory for LVADs, thereby they can provide effective transvalvular flow in a novel method for long-term support via a potential minimally invasive access. The accessory has already been tested *in vivo* [8]. Another group performed off-label transapical LV cannulation and support with promising results in 50 CS patients with dual-lumened devices created originally for right ventricular support. Thirty-day survival was 56% [9].

3D printing in cardiovascular medicine is a rapidly growing field with many possibilities regarding preoperative planning, although future clinical use includes device printing of heart valves, patches or catheters [10]. Currently, clinical utility is limited due to the complex process of device printing, but to create easily modifiable prototypes, especially for a device in progress, it is a useful method. In urgent clinical conditions, as CS, a device with standard sizes quickly available is more preferred; however, for longer-term circulatory support, personalized, 3D printed devices with a biocompatible material might be beneficial for heart failure patients in the future. We created our prototypes using 3D printing technology to have the potential to make them with patient-specific sizes; however, these cannulas can be manufactured using regular medical tubing as well.

Besides the benefit of a quick off-pump minimally invasive insertion, our transapical dual-lumened device has other significant advantages. LV drainage is performed with the outer cannula effectively, hereby LV dilation with its negative consequences can be avoided, and it provides antegrade perfusion via its inner cannula just above the aortic valve. The most frequently used MCS device is peripheral veno-arterial extracorporeal membrane oxygenation (VA-ECMO), which can provide biventricular and pulmonary support, but without LV unloading causes increased LV end-diastolic pressure, LV dilation, increases myocardial oxygen demand, thrombus formation and pulmonary oedema and thereby worsens myocardial recovery and patient outcome. These complications occur due to retrograde flow along with VA-ECMO’s watershed phenomenon [3, 11]. Limb ischaemia, patient immobility and vascular complications are serious drawbacks of femoral cannulation, which further reduce survival rates [12]. Using an oxygenator with VA-ECMO where pulmonary oxygenation is damaged is crucial; however, where isolated LV support is required, an oxygenator in the system increases anticoagulant need and elevate bleeding risks, which is the most serious complication of VA-ECMO. Using a dual-lumened cannula for LV support with a centrifugal pump can function without an oxygenator; therefore, anticoagulant doses can be reduced along with their consequential notable bleeding risk [13]. However, when pulmonary oxygenation worsens, an oxygenator can be added to the system. To avoid the listed complications of sole VA-ECMO's retrograde flow, combined use of available devices are emerging, such as implanting Impella 5.0/5.5 (Abiomed, Danvers, MA, USA) and VA-ECMO together, named ECMELLA, where the Impella is responsible for providing antegrade flow and efficient LV unloading [14]. Inserting Impella 5.5 alone or ECMELLA from different peripheral vascular sites can bridge patients to transplant effectively; however, patient mobility and economic factors are still major issues. Smaller Impellas alone are showing promising results in CS, caused by acute myocardial infarction; however, intravascular haemolysis, cost, durability and flow capability are still significant limiting factors, aside access-site complications, limb ischaemia and bleeding, which affect mortality significantly [3, 15, 16].

### Limitations

It is a limitation that for our feasibility study we used a roller pump instead of a centrifugal pump. However, to test our cannula concept, a roller pump was a cheaper, easily available and adequate choice. With a venous reservoir, the support flow was not inflow-dependent, as it would be with a centrifugal pump. Thereby we could test our cannula’s haemodynamic capability until 5.5 l/min flow. Regarding the appropriate examination of haemolysis, in the future, we plan to use centrifugal pumps with longer study time, now that we confirmed the performance of the cannula *in vivo*. Furthermore, for the experiments we used a small sample size of healthy porcines without LV failure, and for further studies, a left heart failure model would provide us more accurate pathophysiology and information of right ventricular function on support, using additional echocardiography control.

## CONCLUSIONS

The short-term animal experiments demonstrate that by 3D printing technology and earlier CT scan-based planning, our cannula prototypes were adequately insertable to anatomically similar porcines as human dimensions. These cannulas were haemodynamically competent to provide sufficient (up to 5.5 l/min) circulatory support and unloading for the LV of the animals using an extracorporeal pump. Further studies are necessary to examine biocompatibility and the feasibility for longer-term support with a centrifugal pump.

## Supplementary Material

ezaf173_Supplementary_Data

## Data Availability

The datasets used and/or analysed during the current study are available from the corresponding author on reasonable request.

## ABBREVIATIONS

ABP: Arterial blood pressure
CO: Cardiac output
CS: Cardiogenic shock
CVP: Central venous pressure
HR: Heart rate
LV: Left ventricle
LVAD: Left ventricular assist device
MAP: Mean arterial pressure
MCS: Mechanical circulatory support
SAP: Systolic arterial pressure
VA-ECMO: Veno-arterial extracorporeal membrane oxygenation
