# Supplementary material for: Minimally invasive, transapical dual-lumened cannula for short-term left ventricular support in a clinically relevant large animal model
Source: Eur J Cardiothorac Surg. 2025 May 21;67(6):ezaf173. doi: 10.1093/ejcts/ezaf173 (PMC12209791; doi:10.1093/ejcts/ezaf173)
Supplement: ezaf173_Supplementary_Data [file ezaf173_Supplementary_Data.zip › supplementary material 1.docx]

|  | pH  arterial venous | | pO2 (mmHg)  arterial venous | | pCO2 (mmHg)  arterial venous | | HCO3 mmol/l  arterial venous | | BE  arterial venous | |
| --- | --- | --- | --- | --- | --- | --- | --- | --- | --- | --- |
| Baseline (no pump flow) | 7.4±0.12 | 7.36±0.15 | 325.6±115.6 | 64.9±11.2 | 49.5±14.9 | 60.5±20.5 | 28±3 | 30.5±3.3 | 2.5±1.5 | 4.1±0.9 |
| 2.5 l/min | 7.53±0.06 |  | 391.3±46.9 |  | 31.4±6.7 |  | 25.1±2.4 |  | 2.7±1.3 |  |
| 3.5 l/min | 7.48±0.05 |  | 330.9±22 |  | 35.6±7 |  | 25.6±2.7 |  | 2.3±1.7 |  |
| 4 l/min | 7.46±0.04 |  | 313±18.7 |  | 36.1±6.1 |  | 24.8±2.5 |  | 1.2±1.8 |  |
| 4.5 l/min | 7.44±0.04 |  | 300±37.2 |  | 34.8±5.5 |  | 23.4±3.4 |  | -0.4±3.3 |  |
| 5 l/min | 7.44±0.04 |  | 331.7±76.5 |  | 33.8±4.6 |  | 22.7±3 |  | -1.1±2.9 |  |
| 5.5 l/min | 7.45±0.04 | 7.38±0.07 | 298.2±40.9 | 199.4±97.3 | 33.7±4.9 | 40.8±8.2 | 22.8±2.9 | 23.4±3.7 | -1.1±2.7 | -0.8±2.9 |
| Δ 5.5 l/min-baseline  (p value) | 0.04±0.09  (0.39) | 0.02±0.14  (0.63) | 7.9±142  (0.88) | 134.5±11.7  (**0.02**) | -16±12.6  (**0.02**) | -20±14.3  **(0.04**) | -5.7±3.2  **(<0.01**) | -7.1±4.6  (**<0.01**) | -4.1±2.3  (**<0.01**) | -5±4.9  (**<0.01**) |

**Supplementary table 1: Acid-base measurements**

BE=base excess, , p<0.05 values are highlighted; values are expressed as mean ± standard deviation
